# Cell‐free transcriptomic profiles and mechanism insights in female androgenetic alopecia

**DOI:** 10.1002/ctm2.70471

**Published:** 2025-11-14

**Authors:** Lingling Jia, Mingyang Lu, Siwei Deng, Yongcheng Jin, Changjiang Zhao, Ruiyu Luo, Yuan Zhu, Zihan Li, Zixuan An, Hua Jiang, Yufei Li

**Affiliations:** ^1^ Department of Plastic Surgery, Shanghai East Hospital, School of Medicine Tongji University Shanghai China; ^2^ Medical College Tongji University Shanghai China; ^3^ OxTium Technology Co., Ltd Shenzhen Guangdong China; ^4^ Research Institute of Tsinghua University in Shenzhen Shenzhen Guangdong China; ^5^ St Hugh's College University of Oxford Oxford UK

1

Dear Editor,

Our study presents a novel predictive machine learning model that demonstrates the potential of plasma cell‐free RNA (cfRNA) for diagnosing and prognosing female androgenetic alopecia (FAGA). We identified cell‐free *DNAJB9* as significantly associated with FAGA through bioinformatic analysis and machine learning followed by RT‐qPCR validation (Figure 1A).

**FIGURE 1:**
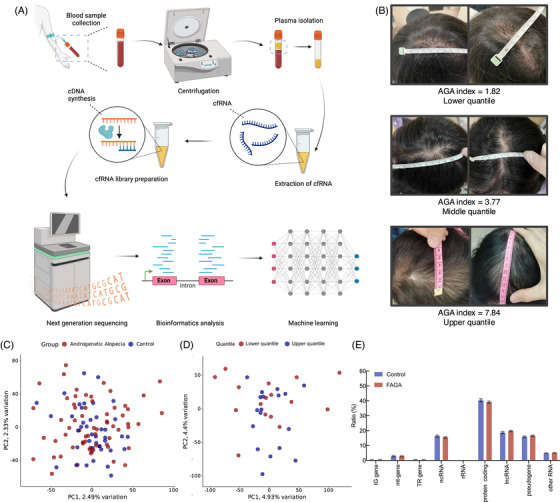
Study overview and sample characteristics. (A) Workflow illustrating blood collection, plasma separation, cfRNA extraction, cDNA library construction, sequencing, and bioinformatics analysis, culminating in the development of a machine learning classification model for FAGA‐associated gene identification. (B) Representative scalp images of FAGA patients, illustrating varying severity levels across lower (index = 1.82), middle (index = 3.77), and upper (index = 7.84) FAGA index quantiles. (C) PCA comparing cfRNA expression profiles between FAGA patients (*n* = 65) and non‐FAGA controls (*n* = 41). (D) PCA comparing cfRNA expression profiles between patients in the upper (*n* = 16) and lower FAGA (*n* = 18) index quartiles. (E) Distribution of RNA types in cfRNA samples from FAGA patients (*n* = 65) and non‐FAGA controls (*n* = 41). Abbreviations: AGA = androgenetic alopecia; PCA = principal component analysis. According to Ensembl Biotypes, RNA biotypes were categorised: IG gene (Immunoglobulin gene); mt‐gene (comprising mt‐rRNA and mt‐tRNA); TR gene (T‐cell receptor gene); ncRNA (including miRNA and miscRNA); rRNA (ribosomal RNA); lncRNA (long noncoding RNA); and other RNA. The ‘other RNA’ category consists of TEC, ribozyme, sRNA, scRNA, scaRNA, snRNA, snoRNA, vault_RNA, and artefact sequences.

FAGA manifests heterogeneously,^1^ often as diffuse thinning of the crown and frontal scalp.^2^ It's pathogenesis critically involves androgen‐hair follicle interactions and WNT and JAK‐STAT signalling.^3^ The cfRNA in bodily fluids have shown diagnostic/prognostic potential for various diseases.^4^ Machine learning is increasingly used to analyse complex cfRNA data.^5^ However, the potential association between cfRNA and FAGA remains unclear.

In this study, we performed high‐throughput RNA sequencing and systematic analysis on plasma samples from 65 FAGA patients and 41 controls (Table S1). The sample inclusion criteria for the FAGA group included sparse hair with gradually shrinking hair follicles (Figure 1B). Exclusion criteria encompassed individuals with congenital sparse hair or other dermatological conditions, as well as those using hair growth medications, antiseptic shampoos, or hair care products. Control participants had no personal or familial history of patterned hair loss, no clinical evidence of hair loss, and no other hair disorders. The severity of androgenetic alopecia was quantified and classified using the FAGA‐Index, where higher values indicate more severe loss (Figure 1B and Table S1), as shown below:

$$I_{FAGA}=\frac{w}{C}\times 100\%,$$

where w indicates hair seam width and C indicates head circumference.

For subsequent analyses comparing disease severity, we focused on the ‘upper’ group as patients in the top 25% of the FAGA‐Index (scores > 5.53) and the ‘lower’ group as those in the bottom 25% (scores < 1.92). Blood test results (Figure S1 and Table S2) showed no significant differences in various haematological and biochemical indicators between the FAGA and control groups. However, testosterone exhibited a significantly lower level in the ‘upper’ group (Figure S5), supporting that the FAGA‐Index effectively enhances the stratification of patients by severity and may facilitate identification of other potential biomarkers in FAGA progression. Greater variation in principal component analysis (PCA) of cfRNA expression profiles between upper and lower FAGA subgroups, compared to that between FAGA and control groups, also suggested increased heterogeneity or molecular diversity within FAGA subtypes (Figure 1C and D).

The RNA biotypes were categorised based on Ensembl classifications with minor adjustments (Figure 1E). Analysis of differentially expressed genes (DEGs) showed that *CYTB*, *RNY1*, and *TMSB4X* were notably upregulated in FAGA patients, whilst *EEF1A1* was significantly downregulated (Figure 2A; Table S5). Furthermore, genes including *ND2*, *ATP6, ND6*, and *PARLP1* exhibited significant expression changes across varying disease severities (Table S7), suggesting their potential association with FAGA progression (Figure 2B). Functional enrichment analysis of these DEGs implicated pathways related to sensory perception, nuclear division, chromosome segregation, and mitosis in FAGA (Figure 2C and D; Tables S6 and S8). Pathway activity analysis reinforced the potential importance of JAK‐STAT and WNTs pathways in FAGA (Figure 3A–C; Tables S17–S19). A comparison of transcription factor (TF) activities between FAGA patients and controls (Figure 3D; Table S9) revealed significantly increased activity of *NCOA3* and *MAX*. However, no significant differences in TF activities were observed between upper‐ and lower‐FAGA subgroups (Table S10). Crucially, we observed a significant negative correlation between cell‐free *DNAJB9* expression and the FAGA‐Index, suggesting that low *DNAJB9* expression may be associated with increased FAGA severity (Table S11). Protein–protein interaction network (PPI) analysis further revealed that in FAGA versus controls, upregulated genes drive endocrine compensation and mitochondrial stress responses, while downregulated genes suppress growth signalling (MET/mTORC1) and RNA metabolism (Figure S2). Comparing upper‐ versus lower‐FAGA, there is increased mitochondrial/endocrine activity alongside impaired translation and calcium homeostasis (Figure S3). These results indicate worsening pathway dysregulation with FAGA progression (Tables S12–S15).

**FIGURE 2:**
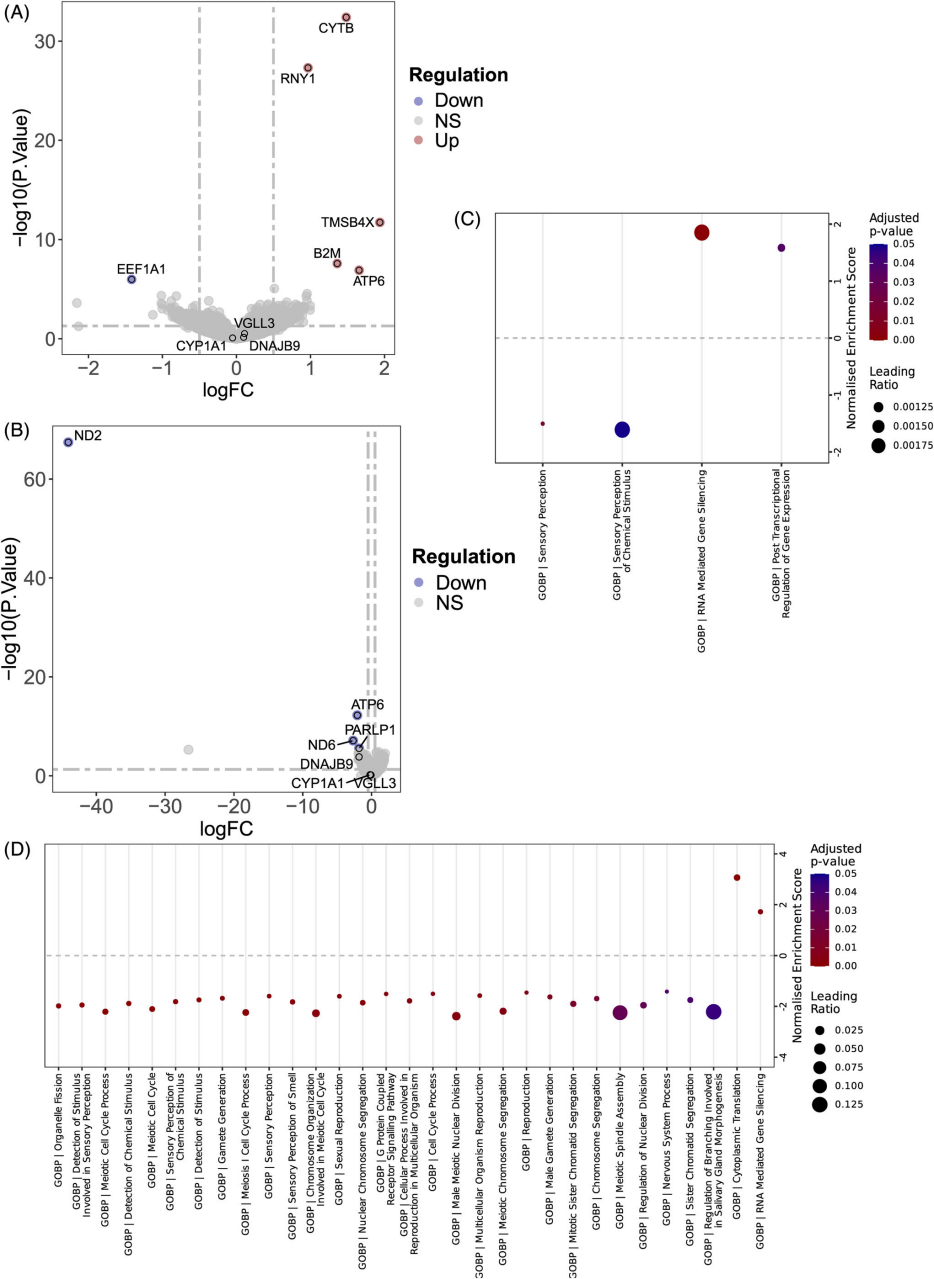
Study workflow and differential expression analysis. (A, B) Volcano plots illustrate the differential expression genes between FAGA (*n* = 65) and non‐FAGA control (*n* = 41) groups (A) and between upper (*n* = 16) and lower FAGA (*n* = 18) quantiles (B). (C, D) Gene set enrichment analysis dot plots illustrate the significantly enriched GOBP terms between FAGA (*n* = 65) and non‐FAGA control (*n* = 41) groups (C) and between upper (*n* = 16) and lower FAGA (*n* = 18) index quantiles (D). Abbreviations: AGA = androgenetic alopecia; FAGA = female androgenetic alopecia.

**FIGURE 3:**
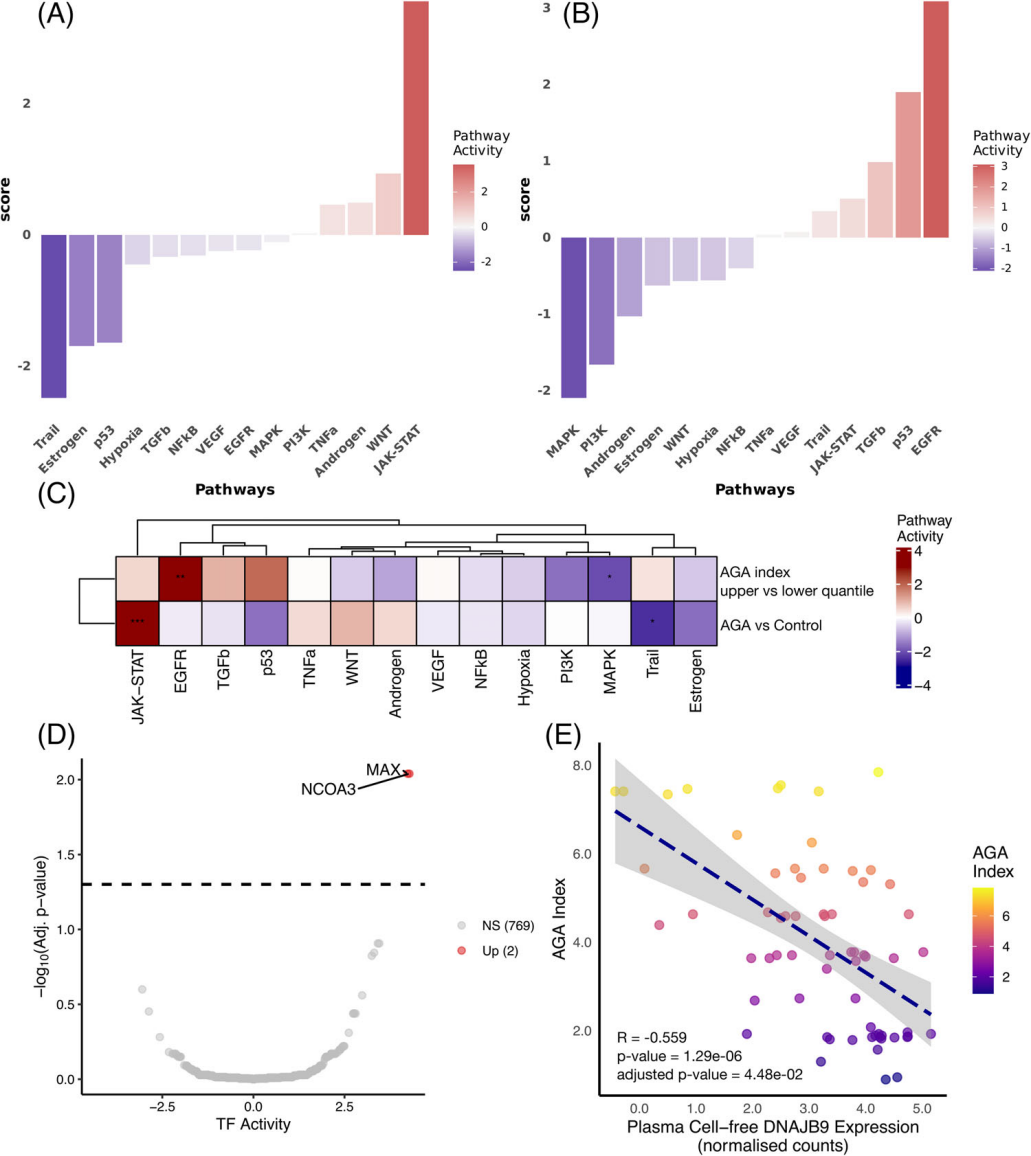
Pathway and transcription factor activity profiling and correlation analysis in FAGA. (A) Pathway activity profiles comparing FAGA patients (*n* = 65) and non‐FAGA controls (*n* = 41). (B) Pathway activity profiles comparing patients in the upper (*n* = 16) and lower FAGA index (*n* = 18) quartiles. (C) Heatmap summary of pathway activity changes. (D) Transcription factor activity profiles comparing FAGA patients (*n* = 65) and non‐FAGA controls (*n* = 41). Transcription factors with adjusted *p*‐values below .05 are highlighted. (E) Correlation between FAGA index and cell‐free *DNAJB9* expression (*n* = 65). Abbreviations: AGA = androgenetic alopecia; FAGA = female androgenetic alopecia.

Subsequently, we developed a predictive model for FAGA using machine learning, employing the GeneLLM downstream classification framework,^6^ a state‐of‐the‐art method for cfRNA classification. After initial feature extraction, deep feature mining was conducted to uncover latent patterns within gene expression profiles indicative of FAGA. The cfRNA RPKM matrix was partitioned into training (40%), validation (40%), and testing (40%) sets (Figure 4A). The large, completely held‐out test set offers a rigorous internal validation of the model's performance on unseen data from the same population. To ensure robustness, hyperparameter optimisation was conducted using 10‐fold cross‐validation. The model achieved an Area Under the Curve (AUC) of .707 for distinguishing FAGA patients from controls (Figure 4B) and .714 for separating high versus low FAGA‐Index scores (Figure 4C). It identified several genes associated with FAGA, including *VGLL3*, *CYP1A1*, antisense to *PDE7B*, and notably *DNAJB9* (Figure 4D). Features correlated with FAGA severity, such as the long non‐coding RNA *ARL14EP‐DT* and pseudogene *TJAP1P1*, were also highlighted (Figure 4E).

**FIGURE 4:**
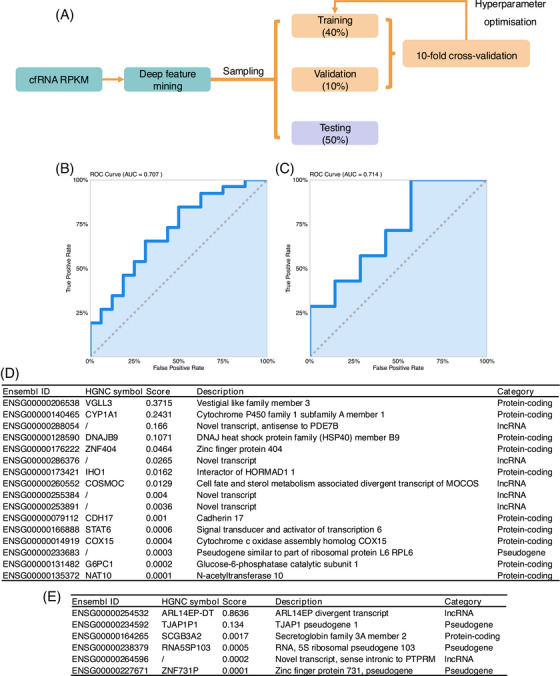
Machine learning model for distinguishing FAGA patients and controls. (A) Model training workflow, including data vectorisation, splitting into training, validation, and testing sets, hyperparameter optimisation with 10‐fold cross‐validation, and performance evaluation. (B, C) ROC curve showing model performance in distinguishing FAGA patients (*n* = 65) from non‐FAGA controls (B) (*n* = 41) and between high (*n* = 16) and low FAGA index (*n* = 18) groups (C). (D) RNA targets identified by the model for distinguishing FAGA patients (*n* = 65) from non‐FAGA controls (*n* = 41). (E) RNA targets identified by the model for differentiating between high (*n* = 16) and low (*n* = 18) FAGA index groups. Abbreviations: AGA = androgenetic alopecia; FAGA = female androgenetic alopecia; ROC = receiver operating characteristic; RPKM = reads per kilobase per million mapped reads.

We performed correlation analysis between the expression levels of the six candidate biomarkers and various blood parameters (Figure S6; Table S20). Nevertheless, the correlation coefficient indicated only weak to moderate associations, implying that more validation is needed between biochemical and molecular diagnosis. Further RT‐qPCR validation in both internal and external cohorts confirmed that cell‐free *VGLL3*, antisense to *PDE7B*, and *DNAJB9* were significantly downregulated in the FAGA patients, while lncRNA *ARL14EP‐DT* and pseudogene *TJAP1P1* were significantly downregulated in the upper FAGA subgroup (Figure S4). Notably, *DNAJB9* is a DNAJ/HSP40 heat shock protein essential for cellular stress responses.^7^ HSP40 family proteins are implicated in regulating androgen receptor (AR) activity, often maintaining AR in an inactive state.^8^, ^9^ Reduced *DNAJB9* expression may potentially disrupt AR signalling in hair follicles, especially under stress.

In summary, as the first investigation integrating cfRNA bioinformatic analysis with machine learning, this study establishes a crucial proof‐of‐concept for the utility of cfRNA in FAGA diagnosis and prognosis, addressing a critical gap in the field and providing a solid foundation for future work. Our exploratory model, whilst moderate in its predictive power, proved effective in highlighting cell‐free *DNAJB9* as a FAGA biomarker and a candidate for therapeutic intervention, meriting further investigation.

## AUTHOR CONTRIBUTIONS

Project supervision was overseen by H.J. Y.L. S.D., Y.J., H.J., and Y.L. were responsible for conceptualisation. L.J., M.L., and Y.L. managed clinical recruitment design. Y.J. designed and conducted the wet‐lab experiments. Data analysis and interpretation were performed by S.D. and Z.A. The primary manuscript writing and revisions were undertaken by L.J., M.L., S.D., and Y.J. Coordination of human samples and data collection was managed by Y.Z., L.J., H.J., M.L., Y.L., Z.L., C.Z., and R.L. All authors contributed to discussions on the results and provided feedback on the manuscript.

## CONFLICT OF INTEREST STATEMENT

No competing interests to disclose.

## CODE AVAILABILITY

All data preprocessing and downstream analyses were conducted using standard bioinformatics tools on a Linux CentOS 8‐based High‐Performance Computing (HPC) system and R version 4.1.3, as provided by OxTium Technology Co. Ltd. Details of the tools, including their names, versions, and specific usage, are outlined in the Methods section. Unless otherwise specified, default parameters were employed for all tools.

## ETHICS STATEMENT

All individuals in this study were thoroughly informed about the objectives, procedure, and possible risks, and written informed consent was obtained before participation. Furthermore, the study protocol received ethical review and approval from the Ethics Review Committee of Shanghai East Hospital (EC.D(BG).016.02.1).

## Supporting information



SUPPORTING INFORMATION

## Data Availability

All the cfRNA sequencing data have been submitted to the NCBI SRA database, accessed under the BioProject accession PRJNA1146172.
