# Supplementary material for: Cell‐free transcriptomic profiles and mechanism insights in female androgenetic alopecia
Source: Clin Transl Med. 2025 Nov 14;15(11):e70471. doi: 10.1002/ctm2.70471 (PMC12617273; doi:10.1002/ctm2.70471)
Supplement: Supplementary file 1 — SUPPORTING INFORMATION [file CTM2-15-e70471-s001.zip › supplemental figures/Figure_S2.pdf]

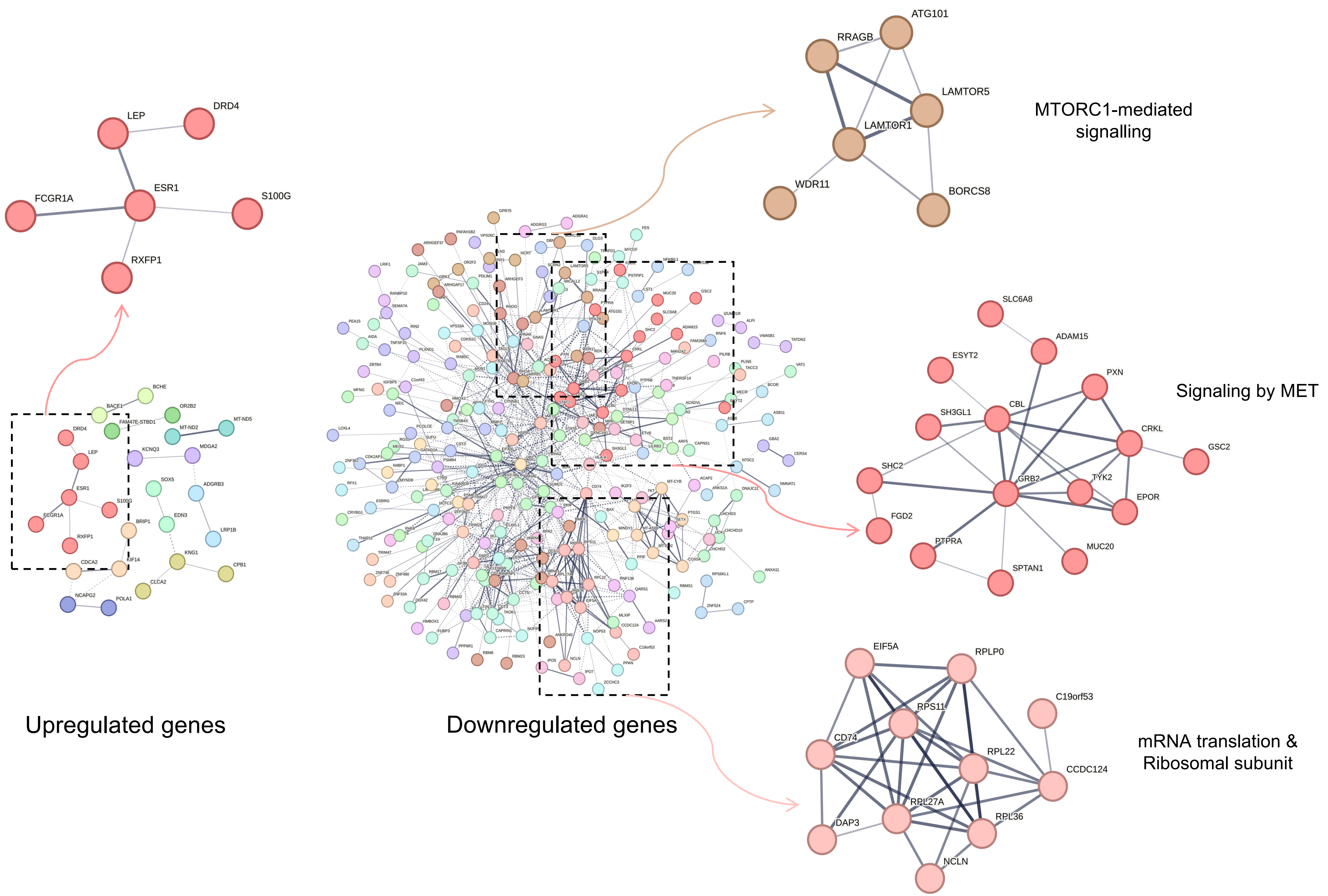
LEP
DRD4
FCGR1A
ESR1
S100G
RXFP1
Upregulated genes
Downregulated genes
ATG101
RRAGB
LAMTOR5
LAMTOR1
WDR11
BORCS8
MTORC1-mediated
signalling
SLC6A8
ADAM15
ESYT2
PXN
Signaling by MET
SH3GL1
CBL
CRKL
GSC2
SHC2
GRB2
TYK2
EPOR
FGD2
PTPRA
SPTAN1
MUC20
EIF5A
RPLP0
RPS11
C19orf53
CD74
RPL22
CCDC124
mRNA translation &
Ribosomal subunit
RPL27A
DAP3
RPL36
NCLN
