# Supplementary material for: Cell‐free transcriptomic profiles and mechanism insights in female androgenetic alopecia
Source: Clin Transl Med. 2025 Nov 14;15(11):e70471. doi: 10.1002/ctm2.70471 (PMC12617273; doi:10.1002/ctm2.70471)
Supplement: Supplementary file 1 — SUPPORTING INFORMATION [file CTM2-15-e70471-s001.zip › supplemental figures/Figure_S6.pdf]

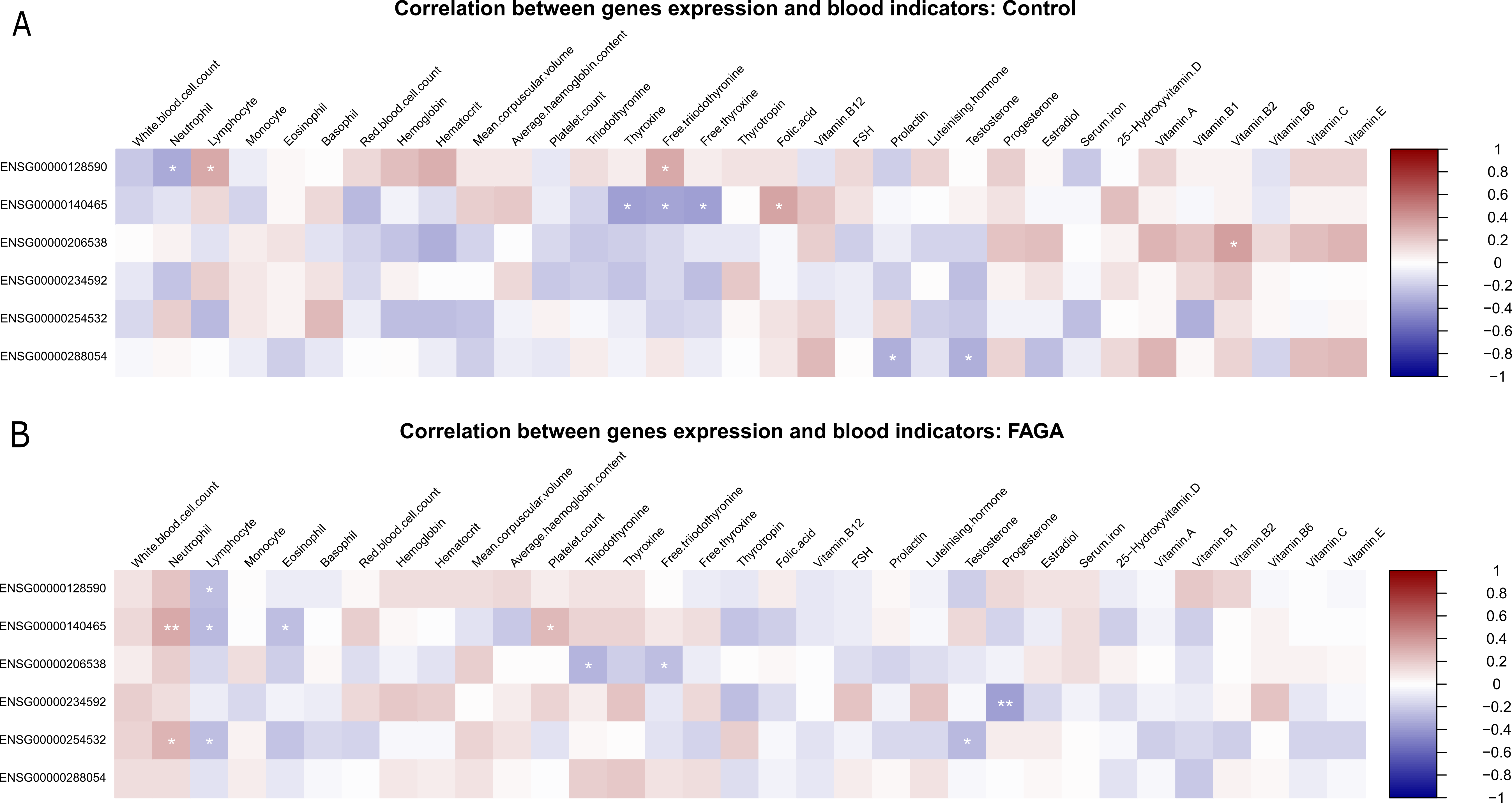
A
Correlation between genes expression and blood indicators: Control
B
Correlation between genes expression and blood indicators: FAGA
White.blood.cell.count
Neutrophil
Lymphocyte
Monocyte
Eosinophil
Basophil
Red.blood.cell.count
Hemoglobin
Hematocrit
Mean.corpuscular.volume
Average.haemoglobin.content
Platelet.count
Triiodothyronine
Thyroxine
Free.triiodothyronine
Free.thyroxine
Thyrotropin
Folic.acid
Vitamin.B12
FSH
Prolactin
Luteinising.hormone
Testosterone
Progesterone
Estradiol
Serum.iron
25−Hydroxyvitamin.D
Vitamin.A
Vitamin.B1
Vitamin.B2
Vitamin.B6
Vitamin.C
Vitamin.E
ENSG00000128590
ENSG00000140465
ENSG00000206538
ENSG00000234592
ENSG00000254532
ENSG00000288054
1
0.8
0.6
0.4
0.2
0
−0.2
−0.4
−0.6
−0.8
−1
