# Supplementary figures and images for: Cell‐free transcriptomic profiles and mechanism insights in female androgenetic alopecia
Source: Clin Transl Med. 2025 Nov 14;15(11):e70471. doi: 10.1002/ctm2.70471 (PMC12617273; doi:10.1002/ctm2.70471)

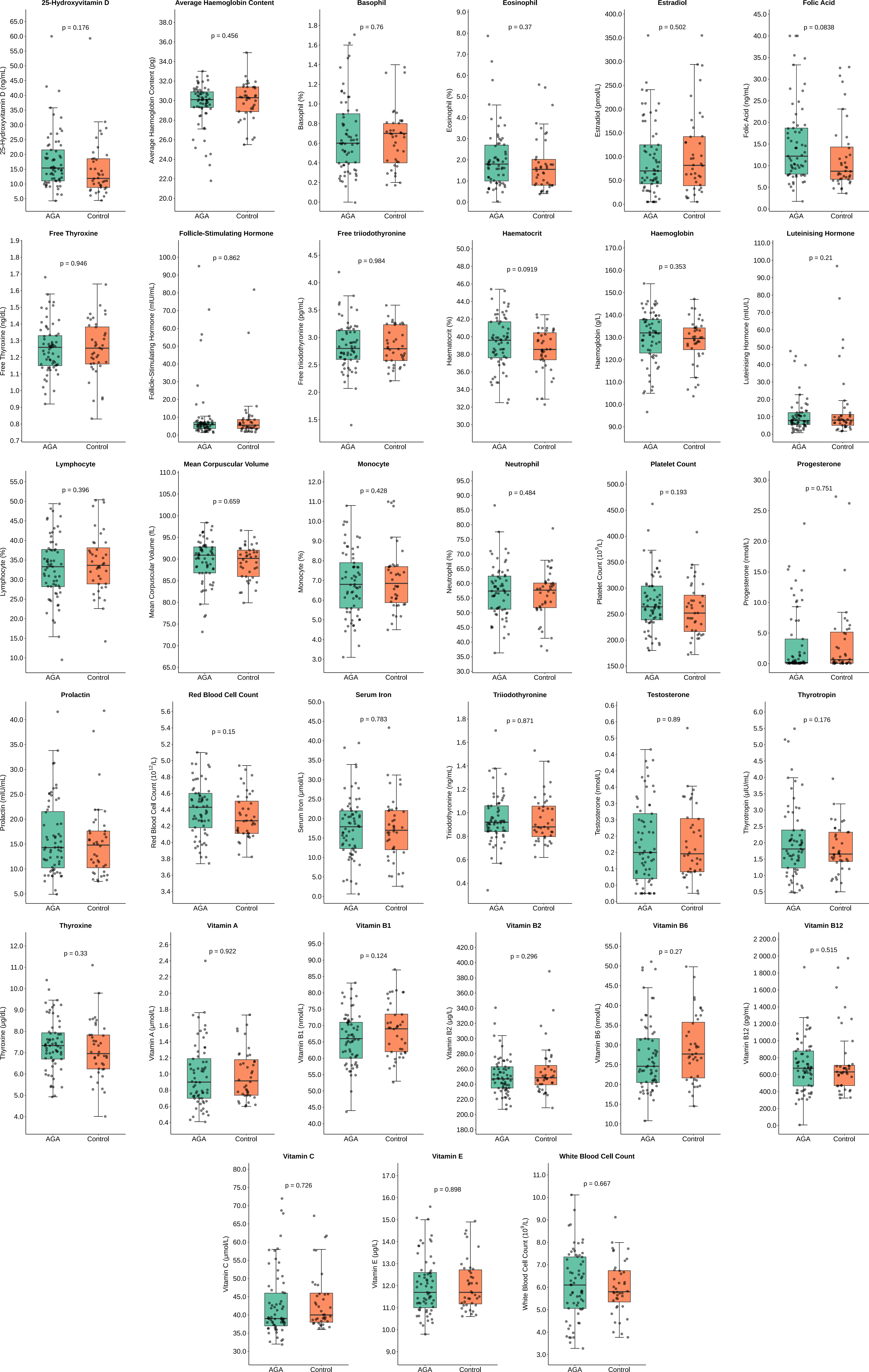

Supplement: Supplementary file 1 — SUPPORTING INFORMATION [file CTM2-15-e70471-s001.zip › supplemental figures/Figure_S1.pdf]

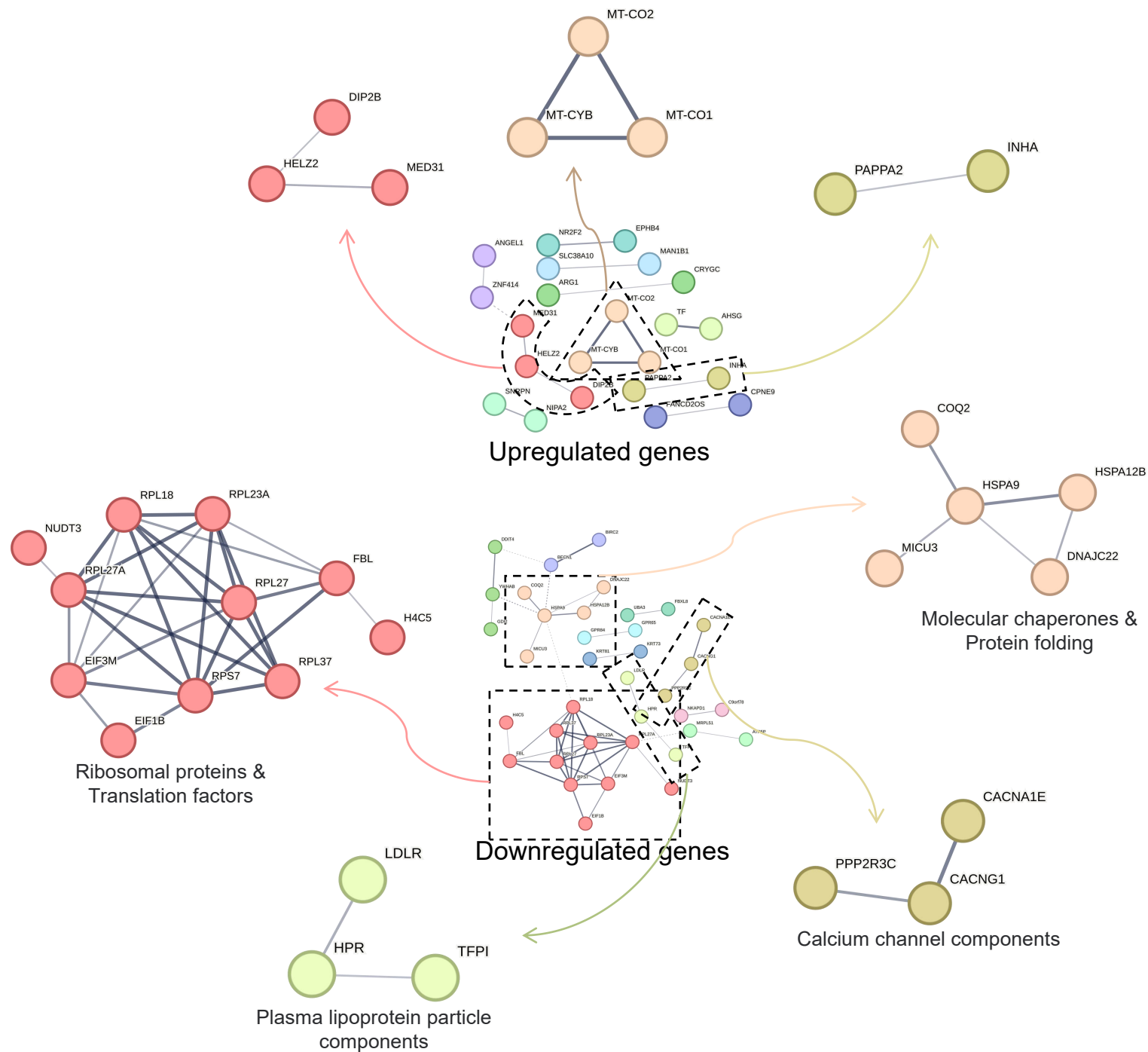

Supplement: Supplementary file 1 — SUPPORTING INFORMATION [file CTM2-15-e70471-s001.zip › supplemental figures/Figure_S3.pdf]

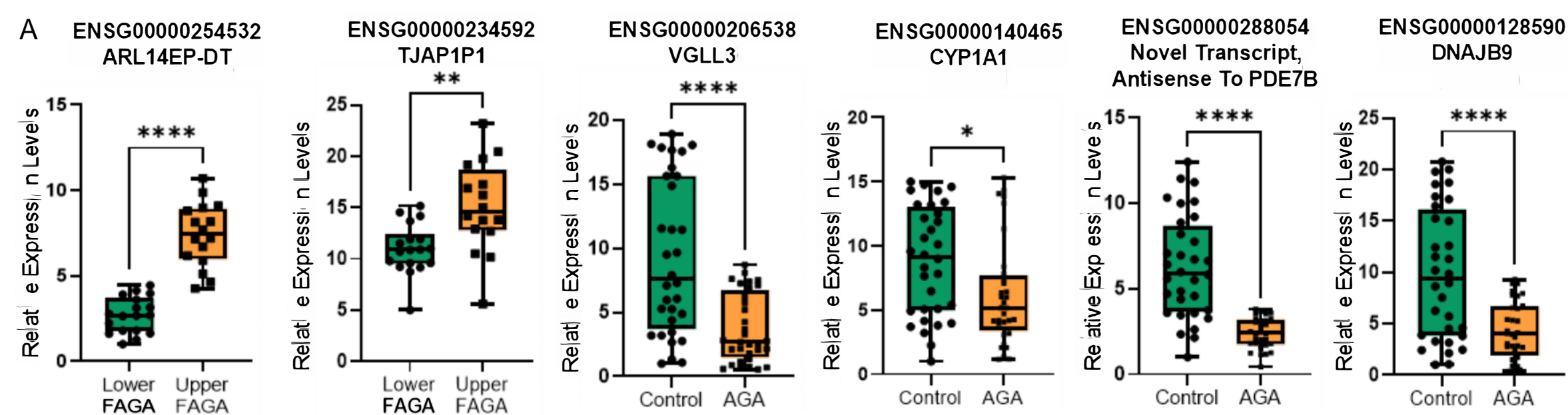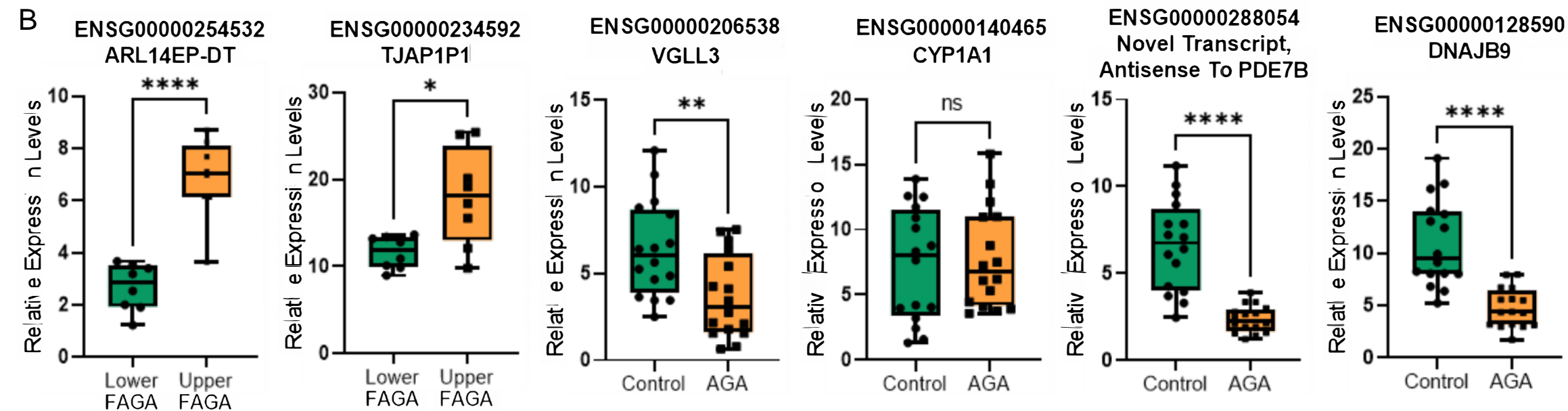

Supplement: Supplementary file 1 — SUPPORTING INFORMATION [file CTM2-15-e70471-s001.zip › supplemental figures/Figure_S4_revised.pdf]

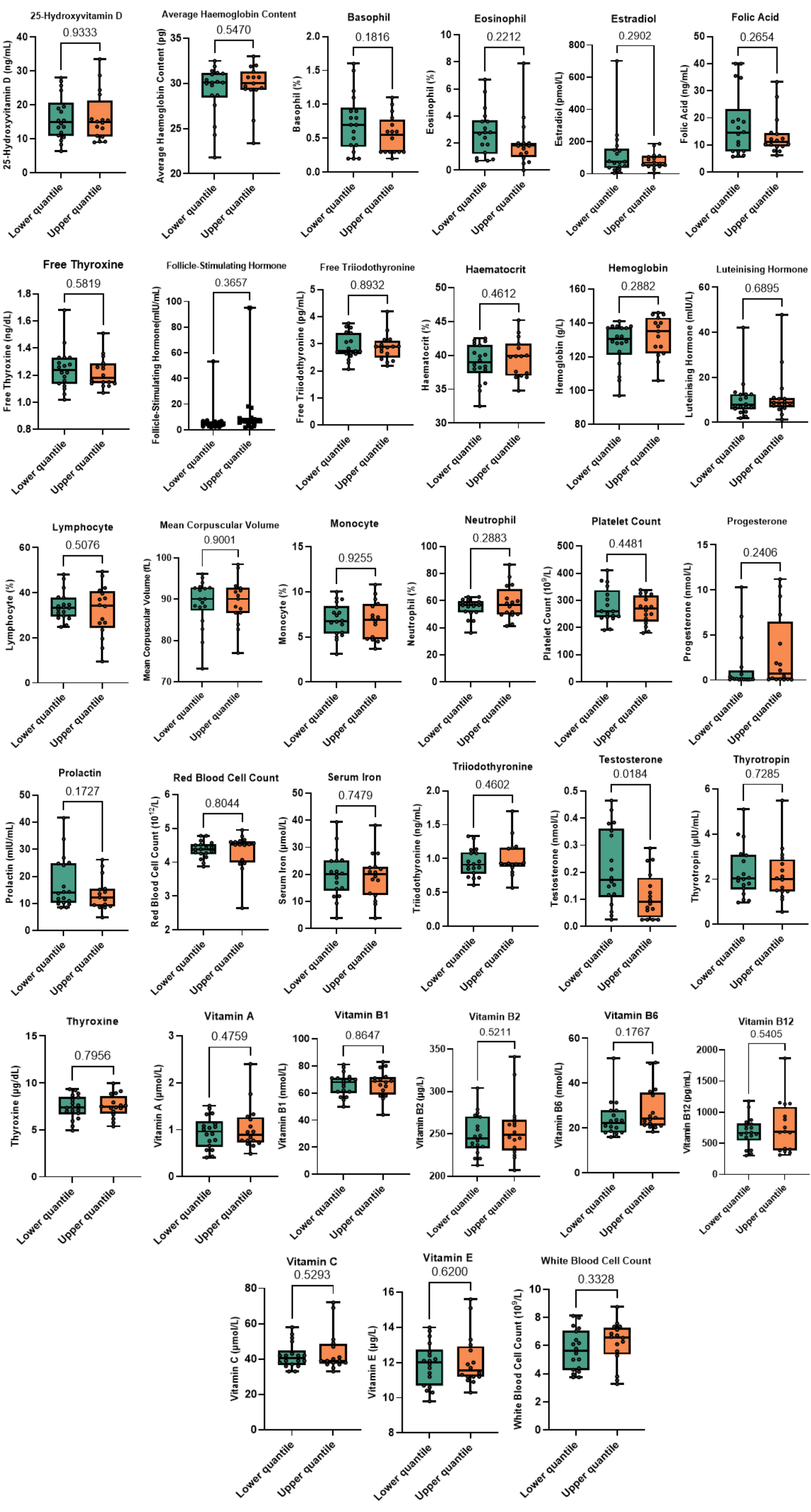

Supplement: Supplementary file 1 — SUPPORTING INFORMATION [file CTM2-15-e70471-s001.zip › supplemental figures/Figure_S5.pdf]
